# The simultaneous presence of demoralization, apathy, and depression has a detrimental impact on both cognitive function and motor symptoms in Parkinson’s disease patients

**DOI:** 10.3389/fpsyt.2024.1345280

**Published:** 2024-02-09

**Authors:** Xiaobo Zhu, Jing Gan, Na Wu, Yu Zhang, Zhenguo Liu

**Affiliations:** ^1^ Department of Neurology, Xinhua Hospital Affiliated to Shanghai Jiao Tong University School of Medicine, Shanghai, China; ^2^ Department of Neurology, QingPu Branch of Zhongshan Hospital Affiliated to Fudan University, Shanghai, China

**Keywords:** Parkinson’s disease, demoralization, apathy, depression, cognitive function, motor symptoms

## Abstract

**Objective:**

Parkinson’s disease (PD) is marked not only by motor symptoms but also by neuropsychiatric manifestations, including demoralization, apathy, and depression. Understanding the clinical distribution and characteristics of these co-occurring symptoms is crucial for improving quality of life of PD patients.

**Methods:**

This study enrolled 195 Chinese PD patients from Xinhua Hospital affiliated to Shanghai Jiao Tong University School of Medicine. The study involved analyzing the clinical characteristics related to the simultaneous presence of demoralization, apathy, and depression in PD patients. Linear regression was employed to elucidate the linear trend between the quantity of negative neuropsychiatric symptoms and cognitive function, as well as motor symptoms and motor complications. SPSS mediation models were utilized to investigate whether the severity of cognitive function mediated the connection between multiple negative neuropsychiatric symptoms and motor symptoms.

**Results:**

Among PD patients, a notable 57.5% experience the presence of multiple concurrent negative neuropsychiatric symptoms. Our investigation unveiled a correlation where patients with more negative neuropsychiatric symptoms displayed heightened cognitive impairment (P=0.048) and more severe motor symptoms (P=0.024), following a linear trend with increasing symptom numbers. Additionally, cognitive impairment played a partial mediating role in the impact of multiple negative neuropsychiatric symptoms on motor symptoms (β=0.747; 95% bootstrap confidence interval: 0.195 to 1.532).

**Conclusions:**

The co-occurrence of these negative neuropsychiatric symptoms has the potential to worsen cognitive function and motor symptoms in PD patients. Moreover, cognitive impairment was identified as playing a partial mediating role in the relationship between multiple negative neuropsychiatric symptoms and motor symptoms.

## Introduction

Neuropsychiatric symptoms are specific non-motor symptoms (NMS) in Parkinson’s disease (PD) and encompass a variety of clinical manifestations. Among these, negative neuropsychiatric symptoms (including depression, apathy, and demoralization) have garnered significant attention. Notably, depression and apathy are reported more frequently in individuals with PD (1, 2). Depression primarily manifests as feelings of emptiness and hopelessness, accompanied by emotional restraint and anhedonia (3). Reports indicate that the prevalence rates of depression vary widely, ranging from 3% to 80%, and this condition often persists over time (3). Depression in PD has shown associations with disease duration, the severity of motor symptoms, the emergence of motor complications or fluctuations, and the use of dopaminergic medication (4). Apathy refers to a diminished motivation and a reduction in goal-directed behaviors, and it can exhibit various features (5). The prevalence of apathy has been observed to range from 16.5% to 62.3%, with an increase noted as the disease progresses (5, 6). In PD, apathy has been independently associated with factors such as low levels of education, cognitive impairment, depression, male gender, and advanced age (7). While both apathy and depression can exhibit symptoms like psychomotor retardation, anhedonia, anergia, reduced physical activity, and decreased enthusiasm for usual interests, apathy is characterized by diminished activities, interests, and emotions. In contrast, depression is defined by the amplification of negative thoughts, beliefs, and emotions (5). The primary distinction between apathy and depression lies primarily in the realm of mood. Apathy is characterized by a neutral mood, whereas depression is distinctly negative and often accompanied by emotional suffering (8). Demoralization in PD has received limited attention, and there have been few studies on this aspect. Demoralization is a psychological phenomenon marked by feelings of helplessness, hopelessness, a sense of failure, and an inability to cope with stressful situations (9). Recent studies have reported varying prevalence rates of demoralization in PD across different populations, ranging from 18.1% to 19.9% (10–12). These studies have demonstrated that several factors may contribute to the risk of demoralization in PD patients, including motor dysfunction, stigma, and perceived challenges with mobility (10, 11). Similar to apathy, demoralization can manifest as reduced motivation, but the distinction lies in the fact that the latter arises from a subjective feeling of incompetence (9). Demoralization and depression are two emotional states that share some similarities yet remain distinct. In depression, the appropriate course of action is known, yet there is decreased motivation. Alternatively, in demoralization, there is prominent uncertainty as to the appropriate course of action (10, 13, 14). Furthermore, additional differences between demoralization and depression, such as changes in appetite and sleep patterns, have also been highlighted (9).

Numerous prior studies have concentrated on isolated subtypes of negative neuropsychiatric symptoms. It is important to note that individuals with PD frequently experience a combination of multiple negative neuropsychiatric symptoms. Nevertheless, there exists a dearth of research exploring and dissecting the clinical distribution and traits of intricate negative neuropsychiatric symptom clusters in PD patients. Negative neuropsychiatric symptoms can be observed across various stages of PD development and can considerably compromise the quality of life. Moreover, the presence of cognitive impairment, the exacerbation of motor symptoms, and the emergence of motor complications serve as significant indicators of disease progression in individuals with PD (15). The relationship between the heightened occurrence of negative neuropsychiatric symptoms and their impact on cognitive function, motor symptoms, and motor complications in PD patients remains largely unexplored. Investigating the influence of negative neuropsychiatric symptoms on cognitive function, motor symptoms, and motor complications could offer valuable insights for implementing early interventions aimed at enhancing the quality of life and extending its duration.

Hence, our study is designed to investigate the clinical distribution and attributes of intricate negative neuropsychiatric symptoms in individuals with PD. Additionally, we aim to examine the correlation between an elevated count of negative neuropsychiatric symptoms and their potential impact on cognitive function, motor symptoms, and motor complications.

## Methods

### Participants

From December 2020 to July 2022, a total of 195 subjects were recruited from the Department of Neurology at Xin Hua Hospital, affiliated with Shanghai Jiaotong University School of Medicine. These patients were diagnosed with Parkinson’s disease based on the Movement Disorder Society (MDS) PD Criteria (16).

The exclusion criteria comprised the following: 1) Secondary Parkinsonism; 2) Patients undergoing deep brain stimulation treatment; 3) Concurrent presence of chronic psychiatric conditions other than depression or anxiety (e.g., schizophrenia); 4) Coexistence of other neurological disorders, including multiple sclerosis, hereditary neuropathy, stroke, Alzheimer’s disease, or brain tumors. A neurologist conducted structured interviews with all included patients to gather demographic, clinical, and pharmacological information. This study was completed in accordance with Helsinki Declaration and ethically approved by the Ethics Committee of XinHua Hospital, which is affiliated with Shanghai Jiao Tong University School of Medicine. All participants provided written informed consent.

### Clinical assessment

Demoralization was evaluated using established questionnaires recognized as gold standards in the field of psychosomatic research: the Demoralization subscale of the Diagnostic Criteria for Psychosomatic Research (DCPR-D) (17) and Kissane Demoralization Scale (KDS) (18). DCPR-D and KDS have been used in previous studies in PD patients to assess demoralization (10). Depression was assessed with the Hamilton Depression Scale(HAMD) (19). Most items on the scale are rated on a 5-point scale from 0 to 4 (0 none, 1 mild, 2 moderate, 3 severe, 4 very severe), and a few items are rated on a 3-point scale from 0 to 2 (0 none, 1 mild-to-moderate, 2 severe), with higher scores indicating more severe depressive symptoms. Depression was defined as HAMD score ≥8. Apathy was assessed with the Short-form Lille Apathy Rating Scale(LARS-s) (20). LARS-s comprises 12 queries in 7 domains, each corresponding to a clinical manifestation of apathy such as “ What do you do during the day? Tell me about your day-to-day life” and the scale’s overall score range from -15 to +15. Apathy was defined as LARS-s core ≥-7.

The following demographic features data were retrieved from subjects: gender, age, age at PD onset, education, and duration of disease. In addition, the Movement Disorders Society Unified Parkinson’s Disease Rating Scale (MDS-UPDRS) (21), Hoehn and Yahr stage(H&Y) and New Freezing of Gait Questionnaire (NFOG) (22) assess patients’ motor symptoms and disease severity. The NFOG consists of an initial item (part I) which was added to allow FOG detection and FOG severity and impact (parts II and III). participants were considered positive for FOG when they met part I and scored ≥1 on parts II and III. Questionnaire for Impulsive-Compulsive Disorders in Parkinson’ s disease (QUIP) (23), Hamilton Anxiety Scale (HAMA) (24), Montreal Cognitive Assessment (MOCA) (25), Minimum Mental State Examination (MMSE) (26), Frontal Assessment Battery (FAB) (27), Epworth Sleepiness Scale (ESS) (28), REM Sleep Behavior Disorder Questionnaire Hong Kong (RBDQ-HK) (29), International Restless Legs Syndrome Study Group (IRLSSG) (30). The symptoms of impulse control behaviors (ICBs) were assessed with QUIP. The QUIP consists of eight items related to ICBs, and the presence of ICBs symptoms was defined by a score ≥1 on any of the eight items. Participants were considered positive for RBD when they scored ≥19 on the RBDQ-HK, which consists of 13 questions ranging from 0 to 100. participants were considered positive for RLS when they have four diagnostic features from the IRLSSG: 1) A desire to move the extremities usually associated with some definable discomfort; 2) Motor restlessness; 3) Worsening of symptoms at rest with at least temporary relief by activity; 4) Worsening of symptoms later in the day or at night. In addition, we included records of patients’ PD drug use (drug type and Total levodopa equivalent daily dose (LEDD) (31), which was calculated according to previously suggested conversion formulae).

### Statistical analysis

Statistical analysis was carried out using SPSS version 26.0. Categorical variables were compared through the χ2 test, while the t-test or Mann-Whitney test was utilized for continuous variables. T-test for normally distributed data, Mann-Whitney U tests for non-normally distributed data, and chi-square for categorical variables. To explore the predictors and risk factors independently associated with the prevalence of depression, apathy, and demoralization, logistic regression models were employed for both univariate and multivariable analyses. Any univariate association with a p-value < 0.10 was integrated into the multivariate model, and then, employing forward stepwise regression, variables were systematically eliminated until the optimal model was obtained. A significance level of p < 0.05 was set, and the odds ratios (OR) were reported alongside their corresponding 95% confidence intervals (95% CI).

Linear regression analysis was employed to assess the linear trends in clinical characteristics concerning the increased count of negative neuropsychiatric symptoms. For continuous variables, and for proportional variables, logistic regression analysis was utilized. Unstandardized regression coefficients (βs) and corresponding 95% confidence intervals (CIs) were calculated within multivariable-adjusted models for cognitive function (MOCA), motor symptoms (MDS-UDPRS III), and motor complications (MDS-UDPRS IV) associated with the number of negative neuropsychiatric symptoms. The multivariable-adjusted models for the associations mentioned above were adjusted for various factors, including gender, age, age of onset, illness duration, education, freezing gait, impulsive-compulsive behavior, anxiety, sleep disturbance, and dopaminergic therapy.

We conducted tests to assess linear trends by incorporating the number of negative neuropsychiatric symptoms (ranging from 0 to 3) as a continuous variable within the models. For mediation analyses, SPSS mediation modeling software and PROCESS (32) were employed to examine whether the severity of cognitive function mediated the associations between multiple co-existing negative neuropsychiatric symptoms and motor symptoms after controlling for age, disease duration, and education.

In the analysis, both the total effect of multiple negative neuropsychiatric symptoms (represented by the count of negative neuropsychiatric symptoms) on motor symptoms and the indirect effect mediated by cognitive function (MOCA) were considered. A bootstrap estimation approach, using 5000 samples, was adopted to quantify the indirect effect (33) along with 95% confidence intervals (CIs). The indirect effect was deemed significant if the 95% CIs did not encompass zero (34). The threshold for statistical significance was set at P < 0.05.

## Results

### Study population and distribution of subtypes of negative neuropsychiatric symptoms

A total of 195 PD patients participated in our study, with 94 (48.21%) of them being female. The mean age was 67.89 (8.74) years old, and the average age at PD onset was 63.74 (9.39) years old. The mean PD disease duration was 50.64 ( ± 44.85) months, and 50.52% of patients had received more than 9 years of education. The MDS-UPDRS scores for parts I, II, III, and IV were 7.19 (5.82), 11.91 (6.82), 26.24 (14.01), and 1.84 (3.31), respectively. The distribution of HY stages was as follows: 21.54% (n = 42) were at stage 1, 47.69% (n = 93) were at stage 2, and 30.77% (n = 60) were at stages 3 to 5. Among the entire cohort, 79.27% of patients were using levodopa, and 52.85% were using dopamine agonists. The mean LEDD was 401.22 (315.17) mg. A comprehensive overview of demographic data is provided in **Table 1**.

**Table 1:** Characteristics of study participants classified by negative symptoms.

Variable	Mean (SD)									
	Total	Demoralization			Apathy			Depression		
		Yes	No	p Value	Yes	No	p Value	Yes	No	p Value
**N**	**195**	**87**	**108**		**111**	**84**		**113**	**82**	
**Gender,female (%)**	48.21	48.28	48.15	0.986	45.05	52.38	0.310	46.02	51.22	0.473
**Age (years)**	67.89(8.74)	68.56(7.80)	67.34(9.44)	0.558a	69.48(7.95)	65.79(9.34)	**0.002a**	68.79(7.74)	66.65(9.88)	0.143a
**Age at PD onset (years)**	63.74(9.39)	64.01(8.24)	63.04(10.25)	0.854a	64.50(8.91)	62.12(9.89)	0.103a	63.88(8.41)	62.90(10.64)	0.709a
**Disease duration (months)**	50.64(44.85)	51.93(41.63)	49.60(47.46)	0.344a	57.44(49.56)	41.65(36.11)	**0.019a**	56.87(46.55)	42.06(36.00)	**0.037a**
**Education,>9 years (%)**	50.52	45.98	54.21	0.254	46.85	55.42	0.237	54.46	45.12	0.199
**MDS-UDPRS part Total score**	47.13(23.50)	54.61(21.28)	41.11(23.56)	**<0.001a**	55.32(23.69)	36.31(18.40)	**<0.001a**	51.57(22.21)	41.02(23.97)	**0.001a**
**MDS-UDPRS part I score**	7.19(5.82)	8.94(6.19)	5.78(5.12)	**<0.001a**	8.97(6.41)	4.83(3.87)	**<0.001a**	8.12(6.12)	5.90(5.15)	**0.005a**
**MDS-UDPRS part II score**	11.91(6.82)	13.61(6.30)	10.55(6.95)	**<0.001a**	13.67(7.00)	9.50(5.84)	**<0.001a**	13.42(6.81)	9.83(6.31)	**<0.001a**
**MDS-UDPRS part III score**	26.24(14.01)	29.57(13.04)	23.56(14.24)	**0.001a**	30.46(14.01)	20.67(11.98)	**<0.001a**	27.92(13.09)	23.93(14.96)	**0.028a**
**MDS-UDPRS part IVscore**	1.84(3.31)	2.48(3.87)	1.32(2.68)	0.066a	2.32(3.92)	1.21(2.12)	0.310a	2.19(3.49)	1.37(2.99)	**0.002a**
**H&Y (%)**				**<0.001**			**<0.001**			**0.003**
**1**	21.54	8.05	32.41		10.81	35.71		13.27	32.93	
**2**	47.69	50.57	45.37		51.35	42.86		50.44	43.90	
**3-5**	30.77	41.38	22.22		37.84	21.43		36.28	23.17	
**FOG(%)**	30.59	33.78	28.13	0.427	34.07	26.58	0.291	36.27	22.06	**0.049**
**ICBs(%)**	6.15	9.20	3.70	0.113	9.91	1.19	**0.012**	8.85	2.44	0.066
**HAMA**	9.11(6.33)	11.74(7.30)	6.99(4.43)	**<0.001a**	10.62(6.85)	7.11(4.94)	**<0.001a**	12.02(6.01)	5.10(4.24)	**<0.001a**
**MOCA**	23.34(4.86)	22.57(5.20)	23.95(4.49)	0.072a	22.15(5.29)	24.90(3.71)	**<0.001a**	23.15(5.11)	24.22(4.40)	0.430a
**MMSE**	26.78(3.44)	26.24(3.92)	27.21(2.96)	0.067a	26.05(3.91)	27.75(2.39)	**0.001a**	26.29(3.87)	27.53(2.47)	0.091a
**FAB**	15.25(3.21)	14.59(3.88)	15.75(2.47)	0.118a	14.35(3.56)	16.28(2.37)	**<0.001a**	14.98(3.48)	15.65(2.73)	0.343a
**ESS**	4.42(3.94)	4.78(4.20)	4.12(3.71)	0.326a	5.71(4.17)	2.85(2.99)	**<0.001a**	5.12(4.17)	3.29(3.26)	**0.004a**
**RBD (%)**	25.31	28.77	22.47	0.359	26.97	23.29	0.592	32.00	14.52	**0.013**
**RLS (%)**	16.67	16.44	16.85	0.944	15.73	17.81	0.724	20.00	11.29	0.148
**LEDD (mg)**	401.22(315.17)	437.56(316.51)	372.01(312.50)	0.158a	452.80(324.82)	334.30(290.62)	**0.011a**	473.35(334.04)	303.59(259.11)	**<0.001a**
Use of classes of PD medications										
**Levodopa**	79.27	83.72	75.70	0.172	85.32	71.43	**0.018**	85.59	70.73	**0.012**
**Dopamine agonists**	52.85	55.81	50.47	0.460	56.88	47.62	0.201	56.76	47.56	0.206

p-value(a) come from Mann-Whitney test.

PD, Parkinson disease; MDS-UPDRS, Movement Disorders Society-Unified Parkinson’s Disease Rating Scale; H&Y, Hoehn and Yahr; FOG, Freezing of Gait; ICBs, Impulse Control Behaviours; HAMA, Hamilton Anxiety Scale; MOCA, Montreal Cognitive Assessment; MMSE, Minimum Mental State Examination; FAB, Frontal Assessment Battery; ESS, Epworth Sleepiness Scale; RBD, Rapid Eye Movement Sleep Behaviour Disorder; RLS, Restless Legs Syndrome; LEDD, Levodopa Equivalent Daily Dose.

Statistically signficant P-values (P < 0.05) are highlighted in bold.

**Figure 1:**
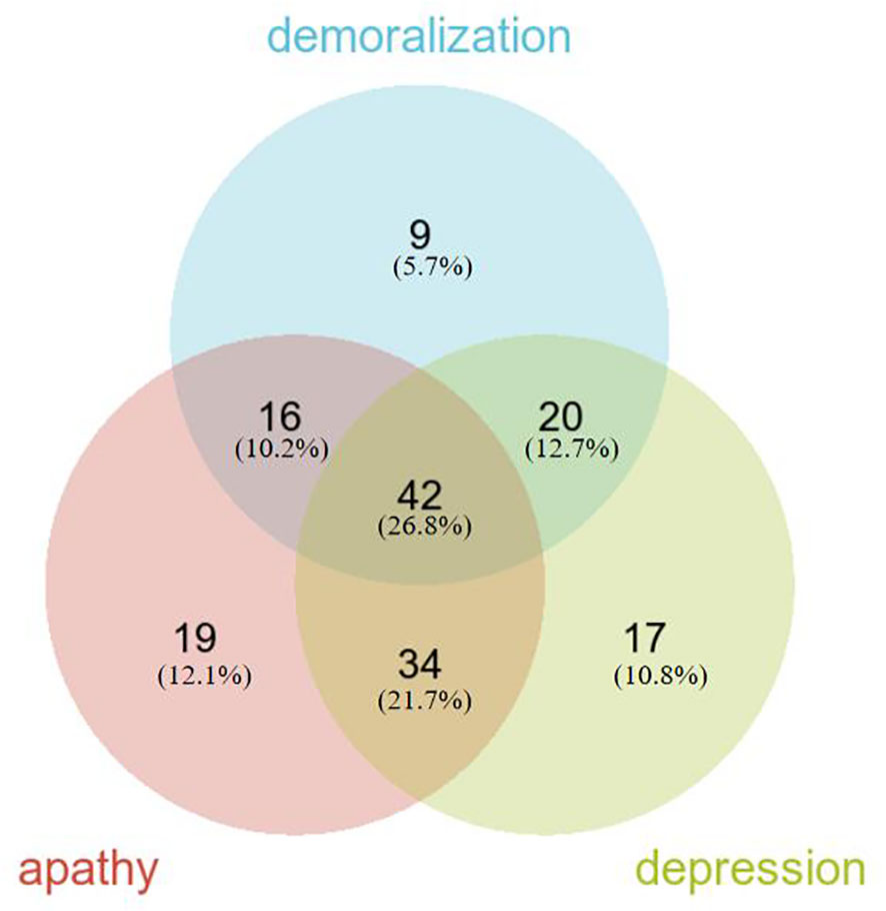
Overlapping prevalence of demoralization, apathy and depression in Parkinson disease.

Regarding negative neuropsychiatric symptoms, the prevalence of demoralization was 44.62% (87/195), apathy was 56.92% (111/195), and depression was 57.95% (113/195). The overlapping prevalence of demoralization, apathy, and depression is depicted in **Figure 1**. The overall prevalence of negative neuropsychiatric symptoms was 80.51% (157/195), with 26.8% (42/157) of patients experiencing a combination of demoralization, apathy, and depression symptoms, which represents the largest proportion. Additionally, 45 patients (23.08%) exhibited only one type of negative neuropsychiatric symptom, while 112 patients (57.44%) had two or more negative neuropsychiatric symptoms (**Figure 1**).

### Factors associated with various subtypes of negative neuropsychiatric symptoms

The characteristics of PD patients in relation to the type of negative neuropsychiatric symptoms (demoralization, apathy, and depression) are presented in **Table 1**. In comparison to patients without negative neuropsychiatric symptoms, those in the demoralization, apathy, and depression groups exhibited higher MDS-UPDRS scores (both total and parts I, II, and III), as well as more severe HY stages and elevated scores on the HAMA. Additionally, a slight increase in disease duration, ESS scores, LEDD, and more frequent use of levodopa were associated with apathy and depression. Furthermore, patients with apathy displayed higher age, a greater prevalence of ICBs, and more pronounced cognitive impairment (as measured by MOCA, MMSE, and FAB). Considering that PD patients usually exhibit specific cognitive function impairments, we further analyzed specific cognitive functions through MOCA. Our findings indicated no significant difference in the total cognitive function (MOCA total score) between PD patients with and without demoralization. However, patients with demoralization exhibited notably poorer performance in the visuospatial/executive domain (p = 0.037). regarding apathy, PD patients with apathy not only displayed significantly lower total cognitive function scores (MOCA total scores) but also manifested impairments in visuospatial/executive (p = 0.008), attention (p = 0.008), language (p = 0.015), delayed recall (p < 0.001), and orientation (p = 0.011). Conversely, PD patients with depression showed no significant difference in total cognitive functioning (MOCA total score) but performed less optimally in the orientation domain (p = 0.023) compared to patients without depression. (**Supplementary Table 1**) Patients experiencing depression also presented with higher MDS-UPDRS part IV scores, an increased occurrence of freezing gait, and a higher frequency of rapid eye movement sleep behavior disorder (RBD). However, no significant associations were observed regarding gender, age at PD onset, education, restless legs syndrome (RLS), or the use of dopamine agonists in relation to the presence of demoralization, apathy, or depression.

Logistic regression analysis, employing a likelihood ratio forward selection, was performed to investigate the associations of demoralization, apathy, and depression. The results demonstrated that for the occurrence of demoralization, contributing factors were H&Y stages 3-5 (OR=4.921, 95% CI: 1.770-13.681, p=0.002) or H&Y stage 2 (OR=2.770, 95% CI: 1.039-7.387, p=0.042), and HAMD score (OR=1.111, 95% CI: 1.049-1.175, p<0.001). For apathy, contributing factors were HAMD score (OR=1.091, 95% CI: 1.030-1.157, p=0.003), FAB score (OR=0.792, 95% CI: 0.687-0.913, p=0.001), and ESS score (OR=1.218, 95% CI: 1.082-1.371, p=0.001). Regarding depression, contributing factors included HAMA score (OR=1.624, 95% CI: 1.378-1.915, p<0.001), LARS-s score (OR=1.102, 95% CI: 1.013-1.198, p=0.024), and LEDD with each increment of 10 points (OR=1.025, 95% CI: 1.008-1.042, p=0.003). (**Supplementary Table 2**).

### Clinical characteristics and factors associated with different numbers of negative neuropsychiatric symptoms

PD patients were classified into four groups based on the number of negative neuropsychiatric symptoms, ranked from 0 to 3, as follows: no negative symptom (n = 38, 19.49%), one type of negative symptom (n = 45, 23.08%), two types of negative neuropsychiatric symptoms (n = 70, 35.90%), and three types of negative neuropsychiatric symptoms (n = 42, 21.54%) (**Table 2**). The clinical attributes of patients were delineated in relation to the quantity of negative neuropsychiatric symptoms, as detailed in **Table 2** and **Supplementary Table 3**.

**Table 2:** Clinical characteristics of PD patients according to number of negative symptoms.

Variable	Mean (SD)				
	Number of Negative symptoms				P-value for trend
	None	One	Two	Three	
**N**	38	45	70	42	
**Gender,female (%)**	60.53	42.22	44.29	50.00	0.413
**Age (years)**	64.29(10.99)	67.29(8.18)	69.70(7.75)	68.76(7.81)	**0.007**
**Age at PD onset (years)**	61.45(12.50)	62.96(7.97)	64.47(8.68)	64.19(8.67)	0.125
**Disease duration (months)**	31.34(35.71)	49.87(31.99)	60.63(51.92)	52.29(47.06)	**0.015**
**Education,>9 years (%)**	50.00	56.82	48.57	47.62	0.619
**MDS-UDPRS Total**	28.00(15.18)	45.42(23.93)	51.06(21.46)	59.74(21.97)	**<0.001**
**MDS-UDPRS I**	3.66(3.11)	6.31(5.12)	7.56(5.56)	10.71(6.80)	**<0.001**
**MDS-UDPRS II**	6.89(3.78)	11.47(7.09)	13.33(6.80)	14.57(6.43)	**<0.001**
**MDS-UDPRS III**	16.68(10.92)	26.13(15.08)	28.40(12.95)	31.40(13.22)	**<0.001**
**MDS-UDPRS IV**	0.76(2.21)	1.51(2.57)	1.91(3.13)	3.05(4.59)	**0.002**
**H&Y (%)**					**<0.001**
**1**	52.63	26.67	11.42	4.76	
**2**	34.21	48.89	54.29	47.62	
**3-5**	13.16	24.44	34.29	47.62	
**FOG(%)**	14.29	32.43	37.50	32.35	0.067
**ICBs(%)**	2.63	2.22	2.86	19.05	**0.004**
**HAMA**	4.05(2.89)	7.11(4.29)	9.52(5.22)	15.10(7.15)	**<0.001**
**MOCA**	25.37(3.74)	23.29(4.25)	23.41(4.87)	21.43(5.68)	**0.001**
**MMSE**	28.21(2.04)	26.80(2.98)	26.89(3.20)	25.29(4.61)	**<0.001**
**FAB**	16.83(1.67)	15.38(2.18)	14.95(3.43)	14.03(4.22)	**<0.001**
**ESS**	2.34(2.50)	3.46(3.00)	5.06(4.27)	6.09(4.31)	**<0.001**
**RBD (%)**	13.79	24.32	25.40	36.36	0.054
**RLS (%)**	13.79	18.92	14.29	21.21	0.626
**LEDD**	244.50(246.34)	372.06(284.66)	455.42(323.19)	487.29(342.05)	**<0.001**
Use of classes of PD medications					
**Levodopa(%)**	63.16	75.56	85.51	87.80	**0.003**
**Dopamine agonists(%)**	42.11	55.56	50.00	61.90	0.116

PD, Parkinson disease; MDS-UPDRS, Movement Disorders Society-Unified Parkinson’s Disease Rating Scale; H&Y, Hoehn and Yahr; FOG, Freezing of Gait; ICBs, Impulse Control Behaviours; HAMA, Hamilton Anxiety Scale; MOCA, Montreal Cognitive Assessment; MMSE, Minimum Mental State Examination; FAB, Frontal Assessment Battery; ESS, Epworth Sleepiness Scale; RBD, Rapid Eye Movement Sleep Behaviour Disorder; RLS, Restless Legs Syndrome; LEDD, Levodopa Equivalent Daily Dose.

Statistically signficant P-values (P < 0.05) are highlighted in bold.

Cognitive impairment, deteriorating motor symptoms, and the presence of motor complications are tightly linked to the progression of PD. Specifically, the associations between cognitive function (MOCA), motor symptoms (MDS-UDPRS III), motor complications (MDS-UDPRS IV), and the number of negative neuropsychiatric symptoms were further adjusted. Upon adjusting for variables such as gender, age, age of onset, disease duration, educational attainment, freezing gait, ICBs, anxiety, sleep disorders (including excessive daytime sleepiness, rapid eye movement sleep behavior disorder, and restless legs syndrome), as well as PD medications, the correlation between the impact of more negative neuropsychiatric symptoms on cognitive function and motor symptoms displayed a slight attenuation (**Table 3**). Meanwhile, the effect on motor complications lost its statistical significance.

**Table 3:** Association between cognitive function, motor symptoms, motor complications and multiple comorbid negative symptoms.

Variable	MOCA score β (95% CI)			MDS-UDPRS III β (95% CI)			MDS-UDPRS IV β (95% CI)		
	Model 1	Model 2	Model 3	Model 1	Model 2	Model 3	Model 1	Model 2	Model 3
number of negative symptoms									
None	1.00 (reference)	1.00 (reference)	1.00 (reference)	1.00 (reference)	1.00 (reference)	1.00 (reference)	1.00 (reference)	1.00 (reference)	1.00 (reference)
One	-2.31 (-4.28to-0.35)	-2.31 (-4.57to-0.05)	-2.09 (-4.35to-0.17)	7.18 (1.72to12.65)	3.31 (-2.51to9.13)	2.84 (-2.95to8.63)	0.04 (-1.11to1.19)	0.11 (-1.10to1.32)	0.10 (-1.08to1.29)
Two	-1.71 (-3.56to-0.14)	-1.48 (-3.74to0.79)	-1.22 (-3.53to1.09)	7.76 (2.62to12.91)	5.83 (-0.01to11.66)	4.53 (-1.38to10.44)	0.11 (-0.98to1.19)	0.20 (-1.01to1.41)	0.23 (-0.98to1.44)
Three	-3.64 (-5.63to1.64)	-3.90 (-6.74to-1.07)	-3.78 (-6.65to-0.91)	11.58 (6.03to17.13)	9.15 (1.83to16.46)	8.56 (1.23to15.89)	1.58 (0.41to2.74)	1.69 (0.17to3.21)	1.51 (0.01to3.01)
P for trend	**0.002**	**0.037**	**0.048**	**<0.001**	**0.011**	**0.024**	**0.011**	0.062	0.079
Increase per disorder	-0.98 (-1.61to-0.35)	-0.96 (-1.86to-0.06)	-0.92 (-1.84to-0.01)	3.42 (1.68to5.16)	2.96 (0.68to5.25)	2.65 (0.35to4.96)	0.48 (-0.11to0.85)	0.46 (-0.02to0.94)	0.43 (-0.05to0.90)

Number of negative symptoms (0–3; demoralization,apathy and depression).

β unstandardized regression coefficient, 95% CI 95% confidence interval, UPDRS Unified Parkinson’s Disease Rating Scale, MOCA.

aβs and 95% CIs were calculated with the use of multivariate linear regression.

Model 1 was adjusted for gender, age, age at PD onset, disease duration, and educational leve.

Model 2 additionally was adjusted for FOG,ICBs, HAMA, ESS, RBD, and RLS.

Model 3 was further adjusted for LEDD and classes of PD medications(Dopamine agonists and Levodopa). Statistically signficant P-values (P < 0.05) are highlighted in bold.

The multivariate regression coefficients (95% CI) for cognitive function (MOCA) compared to the absence of negative neuropsychiatric symptoms were -2.09 (-4.35 to -0.17) for one negative symptom, -1.22 (-3.52 to 1.09) for two negative neuropsychiatric symptoms, and -3.78 (-6.65 to -0.91) for three negative neuropsychiatric symptoms, showcasing a statistically significant trend (trend P = 0.048). Moreover, a similar trend emerged between the severity of motor symptoms and the number of negative neuropsychiatric symptoms (P for trend = 0.035): 2.84 (-2.95 to 8.63) for one negative symptom, 4.53 (-1.38 to 10.44) for two negative neuropsychiatric symptoms, and 8.56 (1.23 to 15.89) for three negative neuropsychiatric symptoms compared to the absence of negative neuropsychiatric symptoms (**Table 3**).

### The mediation analyses of the associations between multiple comorbid negative neuropsychiatric symptoms and motor symptoms

Our findings revealed that an increase in negative neuropsychiatric symptoms can intensify both cognitive impairment and motor symptoms in PD patients. However, whether the impact of negative neuropsychiatric symptoms on these two domains is congruent remains unclear. Consequently, we conducted an in-depth analysis to ascertain if cognitive function mediates the relationship between multiple co-morbid negative neuropsychiatric symptoms and motor symptoms.

**Figure 2:**
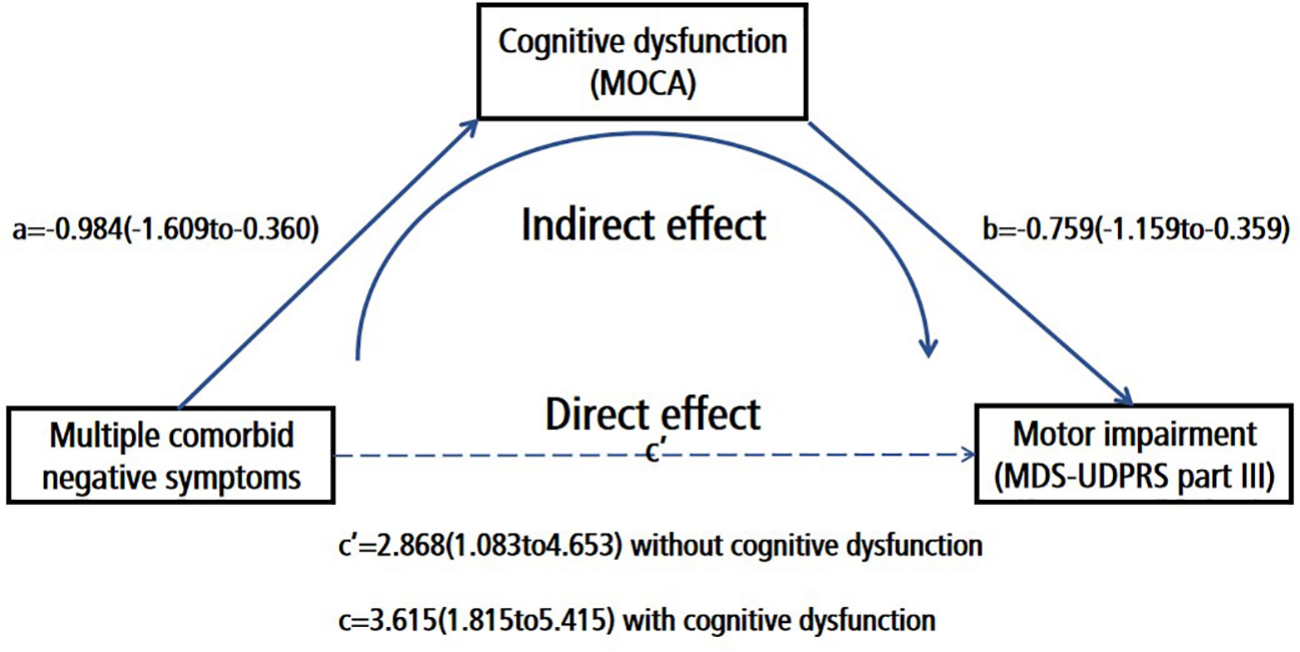
Cognitive dysfunction (MOCA) partially mediated the effect of multiple comorbid negative symptoms on motor impairment (MDS-UDPRS part III); Number of negative symptoms (0–3; demoralization, apathy and depression); Path A Association between the number of negative symptoms and cognitive function; Path B:Association between the number of cognitive function and motor symptoms; Path C:The direct effect of the number of negative symptoms to motor symptoms; Path C The total effect of the number of negative symptoms to motor symptoms.

Our results demonstrated a substantial total effect (β = 3.615; CI: 1.815 to 5.415, p < 0.001) and a noteworthy indirect effect (β = 0.747; 95% boot CI: 0.195 to 1.532) when cognitive function operated as an intermediary between the presence of multiple co-morbid negative neuropsychiatric symptoms and motor symptoms after controlling for age, disease duration, and education. Importantly, the direct effect remained significant (β = 2.868; CI: 1.083 to 4.653, p = 0.002). Thus, we can deduce that cognitive function partially mediates the influence of multiple co-morbid negative neuropsychiatric symptoms on motor symptoms (**Figure 2** and **Supplementary Table 4**). In addition, we further explored whether other variables such as MMSE, ESS, or RBDQ-HK mediated the relationship between multiple comorbid negative symptoms and motor symptoms, and found that MMSE (The indirect effect:β = 0.568; 95% boot CI: 0.053 to 1.336) mediated the relationship between multiple comorbid negative symptoms and motor symptoms, as did MOCA (The indirect effect:β = 0.747; 95% boot CI: 0.195 to 1.532), whereas RBDQ-HK (The indirect effect:β = 0.015; 95% boot CI:-0.369 to 0.468) or ESS (The indirect effect:β = 0.226; 95% boot CI: -0.348 to 0.870) did not. These results are supplemented in **Supplementary Tables 5-7**.

## Discussion

Our study delved into the psychological phenomenon of negative neuropsychiatric symptoms (demoralization, apathy, and depression) within the Chinese PD population. The prevalence of negative neuropsychiatric symptoms among PD patients was found to be notably high, standing at 80.51%. Moreover, a significant portion of patients, specifically 112 individuals (57.44%), exhibited multiple co-morbid negative neuropsychiatric symptoms, whereas a smaller proportion of 45 patients (23.08%) manifested solely one type of negative neuropsychiatric symptom.

Of particular interest is the prevalence of a combined occurrence of demoralization, apathy, and depression, which stood at 21.54%. This figure significantly surpassed the prevalence of any other combination involving two symptoms or the prevalence of any single negative neuropsychiatric symptom. The co-occurrence of depression symptoms alongside apathy (17.44%) was notably more prevalent than any other combination involving two symptoms. In contrast, combinations such as demoralization combined with apathy (8.21%), demoralization combined with depression (10.26%), apathy alone (9.74%), depression alone (8.72%), or demoralization alone (4.62%) were relatively infrequent. Notably, the incidence of demoralization as a standalone symptom was the lowest.

These findings underscore the significance of considering the prevalence of multiple co-morbid negative neuropsychiatric symptoms, which surpasses the occurrence of singular negative symptoms. Hence, when evaluating PD patients who present with negative mood symptom complaints, it is imperative to take into account the potential presence of multiple co-morbidities. A comprehensive assessment and treatment approach are likely to yield more favorable outcomes.

PD patients exhibiting negative neuropsychiatric symptoms (demoralization, apathy, and depression) commonly display an increased burden of both more pronounced motor and non-motor symptoms. Interestingly, our observations also revealed a notable association between demoralization, apathy, and depression with anxiety. This correlation between apathy and depression and anxiety has been corroborated by prior research as well (4, 5). When contrasting patients without apathy to those with apathy, it was discerned that the latter group exhibited older age and more pronounced cognitive impairment. This alignment with previous research findings substantiates our observations (7). It is noteworthy that the presence of apathy was also linked to a higher prevalence of ICBs, which contrasts with prior results (35, 36), yet aligns with recent investigations (37). The interplay between these factors warrants further exploration. Furthermore, patients characterized by apathy or depression demonstrated extended disease durations. This link between disease duration and depression has been documented in previous literature (4), although such a clear association with apathy has not been as pronounced (6). Additionally, patients with apathy or depression exhibited elevated scores on the ESS, indicating increased daytime sleepiness. Notably, REM Sleep Behavior Disorder was more prevalent in PD patients grappling with depression. This suggests a certain interrelation between these negative mood symptoms and distinct sleep disorders. It is also important to acknowledge that a higher LEDD and more frequent usage of Levodopa were observed in the PD subgroup with apathy or depression compared to those without. Consequently, it becomes challenging to definitively deduce whether the link between disease duration and excessive daytime sleepiness is directly correlated with dopamine therapy. Moreover, patients with depression exhibited elevated MDS-UPDRS Part IV scores and an increased incidence of freezing gait, which concurs with earlier investigations (38). In general, it can be deduced that apathy and depression tend to co-occur, possibly indicative of their shared pathogenic role due to similar clinical attributes. In contrast, demoralization seems relatively distinct, hinting at a potentially different underlying pathogenesis.

Given the substantial overlap among the three aforementioned symptoms, we embarked on an exploration of patients exhibiting varying quantities of negative neuropsychiatric symptoms, aiming to unravel their distinct clinical manifestations. This endeavor revealed a noteworthy trend: as the number of negative neuropsychiatric symptoms increased, the severity of both motor and non-motor symptoms escalated in tandem, exhibiting a clear linear progression. Consequently, clinicians are advised to not merely assess the presence of negative neuropsychiatric symptoms, but also scrutinize their specific type and quantity, which could potentially hold critical implications for the trajectory of the patient’s condition.

The confluence of cognitive impairment, exacerbated motor symptoms, and the emergence of motor complications often portends a more ominous disease progression. As such, we concentrated our efforts on scrutinizing the interplay between multiple negative neuropsychiatric symptoms and their impact on cognitive function, motor symptoms, and motor complications. Following meticulous adjustments for various confounding factors, the presence of multiple negative neuropsychiatric symptoms persisted as an independent and substantial contributor to cognitive impairment and the worsening of motor symptoms. Addressing or mitigating the amalgamation of negative neuropsychiatric symptoms in patients could effectively ameliorate cognitive function and motor symptoms, consequently forestalling the march of disease progression. Furthermore, our inquiry delved deeper into the potential associations among the three negative neuropsychiatric symptoms. Specifically, we explored whether the influence of multiple negative neuropsychiatric symptoms on cognitive function and motor symptoms followed parallel pathways. Our findings point towards a mediating role of cognitive function in the relationship between multiple negative neuropsychiatric symptoms and motor symptoms. This mediation was corroborated by a significant indirect effect: cognitive impairment mediates the connection between multiple negative neuropsychiatric symptoms and motor symptoms. While the indirect effect size may be constrained, addressing cognitive impairment could potentially yield substantial enhancements in motor symptoms and overall quality of life, despite the heightened severity of negative neuropsychiatric symptoms. Given the inherent limitations of observational cross-sectional studies, our ability to draw extensive inferences is circumscribed. To provide more robust insights, future longitudinal studies are warranted, as they hold the promise of fortifying our interpretations.

This study boasts several notable strengths that contribute to its significance and potential clinical implications. Foremost, it marks the pioneering endeavor in scrutinizing the clinical attributes and associations of demoralization within a Chinese Parkinson’s disease population. Additionally, it stands as the inaugural clinical analysis to explore the intricate interplay of multiple negative neuropsychiatric symptoms in PD patients. These findings hold crucial implications for clinical practice. Initially, clinicians are urged to recognize the pivotal role those multiple negative neuropsychiatric symptoms, including demoralization, play in the realm of PD. This heightened awareness can pave the way for a more comprehensive assessment, potentially unraveling intricate relationships and informing the development of a holistic treatment approach. Secondly, the study’s revelations underscore the detrimental impact of multiple negative neuropsychiatric symptoms on cognitive impairment and motor symptoms. Notably, the intricate mediating influence of cognitive impairment amidst these symptoms emerges as a key insight. By shedding light on this mediation, clinicians are empowered to better navigate the management of patients, ultimately enhancing their quality of life.

While our study provides valuable insights into the co-occurrence of negative neuropsychiatric symptoms in PD patients and their associations with cognitive and motor function, there are several limitations that warrant consideration: Cross-Sectional Design: One of the primary limitations of this study is its cross-sectional design. As such, it is challenging to establish causality or determine the temporal relationships between the presence of negative neuropsychiatric symptoms, cognitive impairment, and motor symptoms. Longitudinal studies are needed to provide a more comprehensive understanding of these dynamics over time. Selection Bias: Our study was conducted at a single center, which may introduce selection bias. The participants were drawn from Xinhua Hospital, affiliated with Shanghai Jiao Tong University School of Medicine, and may not fully represent the diversity of PD patients in different geographic or clinical settings. Future research should aim for more diverse and representative participant samples. Assessment Tools: Although we employed validated assessment tools for the diagnosis and quantification of negative neuropsychiatric symptoms, cognitive function, and depression, these tools are not without limitations. Variations in the sensitivity and specificity of these instruments may impact the accuracy of our results. Confounding Variables: While we controlled for several potential confounding variables, such as age, gender, disease duration, and medication use, other unmeasured variables (e.g., genetic factors, comorbidities) could contribute to the observed associations. Generalizability: Our study focused on a Chinese PD patient population, and cultural, genetic, or healthcare system factors unique to this population may influence the prevalence and impact of negative neuropsychiatric symptoms. Caution should be exercised when extrapolating our findings to other populations.

## Conclusions

In conclusion, this study’s exploration of demoralization in the context of a Chinese PD population and its analysis of multiple negative neuropsychiatric symptoms represent remarkable strides in our understanding of the condition. The implications for clinical practice are profound, urging clinicians to embrace a more comprehensive perspective in patient evaluation and intervention. The potential to enhance patient care and well-being through nuanced treatment strategies underscores the substantial contributions of this study.

## Data availability statement

The original contributions presented in the study are included in the article/**Supplementary Material**. Further inquiries can be directed to the corresponding authors.

## Ethics statement

The studies involving humans were approved by ethics committee of Xinhua Hospital Affiliated to Shanghai Jiao Tong University School of Medicine. The studies were conducted in accordance with the local legislation and institutional requirements. The participants provided their written informed consent to participate in this study.

## Author contributions

XZ: Conceptualization, Data curation, Formal Analysis, Project administration, Visualization, Writing – original draft. JG: Data curation, Investigation, Software, Supervision, Validation, Writing – original draft. NW: Data curation, Investigation, Software, Supervision, Validation, Writing – original draft. YZ: Conceptualization, Funding acquisition, Methodology, Project administration, Resources, Supervision, Visualization, Writing – review & editing. ZL: Conceptualization, Funding acquisition, Methodology, Project administration, Resources, Software, Supervision, Visualization, Writing – review & editing.

## References

[1] Weintraub D Aarsland D Chaudhuri KR Dobkin RD Leentjens AF Rodriguez-Violante M. The neuropsychiatry of Parkinson's disease: advances and challenges. Lancet Neurol (2022) 21:89–102. doi: 10.1016/S1474-4422(21)00330-6 34942142 PMC8800169

[2] Schapira A Chaudhuri KR Jenner P. Non-motor features of Parkinson disease. Nat Rev Neurosci (2017) 18:435–50. doi: 10.1038/nrn.2017.62 28592904

[3] Ahmad MH Rizvi MA Ali M Mondal AC. Neurobiology of depression in Parkinson's disease: Insights into epidemiology, molecular mechanisms and treatment strategies. Ageing Res Rev (2023) 85:101840. doi: 10.1016/j.arr.2022.101840 36603690

[4] Dissanayaka NN Sellbach A Silburn PA O'Sullivan JD Marsh R Mellick GD. Factors associated with depression in Parkinson's disease. J Affect Disord (2011) 132:82–8. doi: 10.1016/j.jad.2011.01.021 21356559

[5] Pagonabarraga J Kulisevsky J Strafella AP Krack P. Apathy in Parkinson's disease: clinical features, neural substrates, diagnosis, and treatment. Lancet Neurol (2015) 14:518–31. doi: 10.1016/S1474-4422(15)00019-8 25895932

[6] den Brok MGHE van Dalen JW van Gool WA Moll Van Charante EP de Bie RMA Richard E. Apathy in Parkinson's disease: A systematic review and meta-analysis. Mov Disord (2015) 30:759–69. doi: 10.1002/mds.26208 25787145

[7] Marinus J Zhu K Marras C Aarsland D van Hilten JJ. Risk factors for non-motor symptoms in Parkinson's disease. Lancet Neurol (2018) 17:559–68. doi: 10.1016/S1474-4422(18)30127-3 29699914

[8] Richard IH. Depression and apathy in Parkinson's disease. Curr Neurol Neurosci Rep (2007) 7:295–301. doi: 10.1007/s11910-007-0045-z 17618535

[9] Tecuta L Tomba E Grandi S Fava GA. Demoralization: a systematic review on its clinical characterization. Psychol Med (2015) 45:673–91. doi: 10.1017/S0033291714001597 25032712

[10] Koo BB Chow CA Shah DR Khan FH Steinberg B Derlein D. Demoralization in parkinson disease. Neurol (2018) 90:e1613–17. doi: 10.1212/WNL.0000000000005425 PMC5931805 29618626

[11] Zhu B Kohn R Patel A Koo BB Louis ED de Figueiredo JM. Demoralization and quality of life of patients with parkinson disease. Psychother Psychosom (2021) 90:415–21. doi: 10.1159/000514270 33601384

[12] Elfil M Ahmed N Alapati A Bahekar R Kandil M Kim C. Suicidal risk and demoralization in Parkinson disease. J Neurol (2020) 267:966–74. doi: 10.1007/s00415-019-09632-2 31802218

[13] de Figueiredo JM. Depression and demoralization: phenomenologic differences and research perspectives. Compr Psychiatry (1993) 34:308. doi: 10.1016/0010-440X(93)90016-W 8306640

[14] Mangelli L Fava GA Grandi S Grassi L Ottolini F Porcelli P. Assessing demoralization and depression in the setting of medical disease. J Clin Psychiatry (2005) 66:391–94. doi: 10.4088/jcp.v66n0317 15766307

[15] Post B Merkus MP de Haan RJ Speelman JD. Prognostic factors for the progression of Parkinson's disease: A systematic review. Mov Disord (2007) 22:1839–51. doi: 10.1002/mds.21537 17595026

[16] Postuma RB Berg D Stern M Poewe W Olanow CW Oertel W. MDS clinical diagnostic criteria for Parkinson's disease. Mov Disord (2015) 30:1591–601. doi: 10.1002/mds.26424 26474316

[17] Fava GA Freyberger HJ Bech P Christodoulou G Sensky T Theorell T. Diagnostic criteria for use in psychosomatic research. Psychother Psychosom (1995) 63:1–08. doi: 10.1159/000288931 7740096

[18] Kissane DW Wein S Love A Lee XQ Kee PL Clarke DM. The Demoralization Scale: a report of its development and preliminary validation. J Palliat Care (2004) 20:269–76. doi: 10.1177/082585970402000402 15690829

[19] Frank E Prien RF Jarrett RB Keller MB Kupfer DJ Lavori PW. Conceptualization and rationale for consensus definitions of terms in major depressive disorder: remission, recovery, relapse, and recurrence. Arch Gen Psychiatry (1991) 48:851–55. doi: 10.1001/archpsyc.1991.01810330075011 1929776

[20] Dujardin K Sockeel P Carette AS Delliaux M Defebvre L. Assessing apathy in everyday clinical practice with the short-form Lille Apathy Rating Scale. Mov Disord (2013) 28:2014–19. doi: 10.1002/mds.25584 23836341

[21] Goetz CG Tilley BC Shaftman SR Stebbins GT Fahn S Martinez-Martin P. Movement Disorder Society-sponsored revision of the Unified Parkinson's Disease Rating Scale (MDS-UPDRS): scale presentation and clinimetric testing results. Mov Disord (2008) 23:2129–70. doi: 10.1002/mds.22340 19025984

[22] Nieuwboer A Rochester L Herman T Vandenberghe W Emil GE Thomaes T. Reliability of the new freezing of gait questionnaire: agreement between patients with Parkinson's disease and their carers. Gait Posture (2009) 30:459–63. doi: 10.1016/j.gaitpost.2009.07.108 19660949

[23] Weintraub D Mamikonyan E Papay K Shea JA Xie SX Siderowf A. Questionnaire for impulsive-compulsive disorders in parkinson's disease-rating scale. Mov Disord (2012) 27:242–47. doi: 10.1002/mds.24023 PMC3537263 22134954

[24] Thompson E. Hamilton rating scale for anxiety (HAM-A). Occup Med (Lond) (2015) 65:601. doi: 10.1093/occmed/kqv054 26370845

[25] Nasreddine ZS Phillips NA Bedirian V Charbonneau S Whitehead V Collin I. The Montreal Cognitive Assessment, MoCA: a brief screening tool for mild cognitive impairment. J Am Geriatr Soc (2005) 53:695–99. doi: 10.1111/j.1532-5415.2005.53221.x 15817019

[26] Folstein MF Folstein SE McHugh PR. "Mini-mental state". A practical method for grading the cognitive state of patients for the clinician. J Psychiatr Res (1975) 12:189–98. doi: 10.1016/0022-3956(75)90026-6 1202204

[27] Lima CF Meireles LP Fonseca R Castro SL Garrett C. The Frontal Assessment Battery (FAB) in Parkinson's disease and correlations with formal measures of executive functioning. J Neurol (2008) 255:1756–61. doi: 10.1007/s00415-008-0024-6 18821046

[28] Johns MW. A new method for measuring daytime sleepiness: the Epworth sleepiness scale. Sleep (New York N.Y.) (1991) 14:540. doi: 10.1093/sleep/14.6.540 1798888

[29] Shen SS Shen Y Xiong KP Chen J Mao CJ Huang JY. Validation study of REM sleep behavior disorder questionnaire-Hong Kong (RBDQ-HK) in east China. Sleep Med (2014) 15:952–58. doi: 10.1016/j.sleep.2014.03.020 24938584

[30] Walters AS LeBrocq C Dhar A Hening W Rosen R Allen RP. Validation of the International Restless Legs Syndrome Study Group rating scale for restless legs syndrome. Sleep Med (2003) 4:121–32. doi: 10.1016/s1389-9457(02)00258-7 14592342

[31] Tomlinson CL Stowe R Patel S Rick C Gray R Clarke CE. Systematic review of levodopa dose equivalency reporting in Parkinson's disease. Mov Disord (2010) 25:2649–53. doi: 10.1002/mds.23429 21069833

[32] Igartua JJ Hayes AF. Mediation, moderation, and conditional process analysis: concepts, computations, and some common confusions. Span J Psychol (2021) 24:e49. doi: 10.1017/SJP.2021.46 35923144

[33] Shrout PE Bolger N. Mediation in experimental and nonexperimental studies: new procedures and recommendations. Psychol Methods (2002) 7:422–45. doi: 10.1037/1082-989X.7.4.422 12530702

[34] Preacher KJ Hayes AF. SPSS and SAS procedures for estimating indirect effects in simple mediation models. Behav Res Methods Instrum Comput (2004) 36:717–31. doi: 10.3758/bf03206553 15641418

[35] Palmeri R Corallo F Bonanno L Currò S Merlino P Di Lorenzo G. Apathy and impulsiveness in Parkinson disease: Two faces of the same coin? Med (Baltimore) (2022) 101:e29766. doi: 10.1097/MD.0000000000029766 PMC9239641 35776985

[36] Sierra M Carnicella S Strafella AP Bichon A Lhommée E Castrioto A. Apathy and impulse control disorders: yin & Yang of dopamine dependent behaviors. J Parkinson's Disease (2015) 5:625–36. doi: 10.3233/JPD-150535 25870025

[37] Scott BM Eisinger RS Burns MR Lopes J Okun MS Gunduz A. Co-occurrence of apathy and impulse control disorders in Parkinson disease. Neurol (2020) 95:e2769–80. doi: 10.1212/WNL.0000000000010965 PMC7734726 33004605

[38] Cong S Xiang C Zhang S Zhang T Wang H Cong S. Prevalence and clinical aspects of depression in Parkinson’s disease: A systematic review and meta−analysis of 129 studies. Neurosci Biobehav Rev (2022) 141:104749. doi: 10.1016/j.neubiorev.2022.104749 35750224

