# Supplementary material for: The simultaneous presence of demoralization, apathy, and depression has a detrimental impact on both cognitive function and motor symptoms in Parkinson’s disease patients
Source: Front Psychiatry. 2024 Feb 9;15:1345280. doi: 10.3389/fpsyt.2024.1345280 (PMC10884111; doi:10.3389/fpsyt.2024.1345280)
Supplement: Supplementary file 1 [file DataSheet_1.doc]

| **Supplement Table 1 cognitive function of participants classified by negative symptoms** | | | | | | | | | | |
| --- | --- | --- | --- | --- | --- | --- | --- | --- | --- | --- |
| **Variable** | **Mean (SD)** | | | | | | | | | |
|  | **Total** | **Demoralization** | | | **Apathy** | | | **Depression** | | |
|  |  | **Yes** | **No** | **p Value** | **Yes** | **No** | **p Value** | **Yes** | **No** | **p Value** |
| **N** | **195** | **87** | **108** |  | **111** | **84** |  | **113** | **82** |  |
| **MOCA** | 23.34(4.86) | 22.57(5.20) | 23.95(4.49) | 0.072a | 22.15(5.29) | 24.90(3.71) | **<0.001a** | 23.15(5.11) | 24.22(4.40) | 0.430a |
| **Visuospatial/executive** | 3.40(1.25) | 3.15(1.40) | 3.60(1.08) | **0.037a** | 3.17(1.34) | 3.70(1.05) | **0.008a** | 3.37(1.24) | 3.44(1.27) | 0.672a |
| **Naming** | 2.74(0.56) | 2.79(0.49) | 2.69(0.62) | 0.331a | 2.75(0.55) | 2.73(0.59) | 0.899a | 2.73(0.44) | 2.71(0.68) | 0.612a |
| **Attentiom** | 5.28(1.07) | 5.14(1.13) | 5.40(1.01) | 0.050a | 5.07(1.26) | 5.56(1.68) | **0.008a** | 5.12(1.24) | 5.50(0.74) | 0.095a |
| **Language** | 2.41(0.87) | 2.43(0.90) | 2.39(0.84) | 0.557a | 2.27(0.93) | 2.58(0.73) | **0.015a** | 2.44(0.84) | 2.35(0.89) | 0.464a |
| **Abstraction** | 1.63(0.61) | 1.55(0.62) | 1.69(0.59) | 0.072a | 1.57(0.64) | 1.71(0.55) | 0.070a | 1.60(0.61) | 1.67(0.61) | 0.249a |
| **Delayed recall** | 2.28(1.59) | 2.06(1.50) | 2.45(1.64) | 0.091a | 1.90(1.58) | 2.77(1.46) | **<0.001a** | 2.28(1.59) | 2.39(1.65) | 0.474a |
| **Orientation** | 5.60(0.79) | 5.49(0.93) | 5.69(0.65) | 0.193a | 5.45(0.95) | 5.80(0.43) | **0.011a** | 5.48(0.92) | 5.77(0.53) | **0.023a** |

p-value(a) come from Mann-Whitney test

MOCA= Montreal Cognitive Assessment

| **Supplementary Table 2 Logistic regression analysis of different types of negative symptoms** | | | |
| --- | --- | --- | --- |
|  | **OR** | **95% CI** | **P Value** |
| **Demoralization** |  |  |  |
| **H&Y** |  |  |  |
| **1** | 1.000(reference) |  |  |
| **2** | 2.770 | 1.039,7.387 | **0.042** |
| **3-5** | 4.921 | 1.770,13.681 | **0.002** |
| **HAMD** | 1.111 | 1.049,1.175 | **<0.001** |
| **Apathy** |  |  |  |
| **HAMD** | 1.091 | 1.030,1.157 | **0.003** |
| **FAB** | 0.792 | 0.687,0.913 | **0.001** |
| **ESS** | 1.218 | 1.082,1.371 | **0.001** |
| **Depression** |  |  |  |
| **HAMA** | 1.624 | 1.378,1.915 | **<0.001** |
| **LARS-s** | 1.102 | 1.013,1.198 | **0.024** |
| **LEDD(perΔ10 pts)** | 1.025 | 1.008,1.042 | **0.003** |

H&Y=Hoehn and Yahr; HAMD=Hamilton Depression Scale; FAB=Frontal Assessment Battery; ESS=Epworth Sleepiness Scale; HAMA= Hamilton Anxiety Scale; LARS-s=Short-form Lille Apathy Rating Scale; LEDD= levodopa equivalent daily dose;

| **Supplement Table 3 cognitive function of PD patients according to number of negative symptoms** | | | | | |
| --- | --- | --- | --- | --- | --- |
| **Variable** | **Mean (SD)** | | | | |
|  | **Number of Negative symptoms** | | | | **P-value**  **for trend** |
|  | **None** | **One** | **Two** | **Three** |  |
| **N** | 38 | 45 | 70 | 42 |  |
| **MOCA** | 25.37(3.74) | 23.29(4.25) | 23.41(4.87) | 21.43(5.68) | **0.001** |
| **Visuospatial/executive** | 3.79(1.12) | 3.49(1.08) | 3.37(1.19) | 3.00(1.51) | **0.005** |
| **Naming** | 2.66(0.67) | 2.62(0.68) | 2.79(0.48) | 2.86(0.42) | **0.044** |
| **Attentiom** | 5.66(0.53) | 5.38(0.89) | 5.31(1.12) | 4.79(1.35) | **<0.001** |
| **Language** | 2.53(0.76) | 2.33(0.93) | 2.43(0.81) | 2.33(0.98) | 0.471 |
| **Abstraction** | 1.79(0.58) | 1.60(0.54) | 1.66(0.63) | 1.48(0.63) | **0.049** |
| **Delayed recall** | 3.03(1.03) | 2.04(1.58) | 2.36(1.53) | 1.71(1.50) | **0.002** |
| **Orientation** | 5.84(0.37) | 5.82(0.39) | 5.49(0.85) | 5.33(1.12) | **<0.001** |

MOCA= Montreal Cognitive Assessment

| **Supplement Table 4 Mediation model of cognitive dysfunction (MOCA) mediating the effects of multiple comorbid negative symptoms on movement disorders (MDS-UDPRS Part III) after controlling for age, disease duration, and education** | | | | | |
| --- | --- | --- | --- | --- | --- |
|  | **Path Coefficients*** | | | | |
|  | **Path a** | **Path b** | **Path ab** | **Path c’** | **Path c** |
| **number of negative symptoms** | β=-0.984;  95%(-1.609to-0.360);  SE=0.317;  P=0.002; | β=-0.759;  95%(-1.159to-0.359); SE=0.203;  p<0.001; | β=0.747;  95%(0.195to1.532);  Boot SE=0.346; | β=2.868;  95%(1.083to4.653);  SE=0.905;  P=0.002; | β=3.615; 95%(1.815to5.415);  SE=0.913;  p<0.001; |

Number of negative symptoms (0–3; demoralization,apathy and depression);

Path a ：Association between the number of negative symptoms and cognitive function；

Path b ：Association between the number of cognitive function and motor symptoms；

Path ab ：The indirect effect of the number of negative symptoms to motor symptoms；

Path c’：The direct effect of the number of negative symptoms to motor symptoms；

Path c ：The total effect of the number of negative symptoms to motor symptoms；

| **Supplement Table 5 Mediation model of cognitive dysfunction (MMSE) mediating the effects of multiple comorbid negative symptoms on movement disorders (MDS-UDPRS Part III) after controlling for age, disease duration, and education** | | | | | |
| --- | --- | --- | --- | --- | --- |
|  | **Path Coefficients*** | | | | |
|  | **Path a** | **Path b** | **Path ab** | **Path c’** | **Path c** |
| **number of negative symptoms** | β=-0.720;  95%(-1.166to-0.274);  SE=0.226;  P=0.002; | β=-0.788;  95%(-1.358to-0.218); SE=0.289;  P=0.007; | β=0.568;  95%(0.053to1.336);  Boot SE=0.335; | β=3.047;  95%(1.230to4.864);  SE=0.921;  P=0.001; | β=3.615; 95%(1.815to5.415);  SE=0.913;  p<0.001; |

Number of negative symptoms (0–3; demoralization,apathy and depression);

Path a ：Association between the number of negative symptoms and cognitive function；

Path b ：Association between the number of cognitive function and motor symptoms；

Path ab ：The indirect effect of the number of negative symptoms to motor symptoms；

Path c’：The direct effect of the number of negative symptoms to motor symptoms；

Path c ：The total effect of the number of negative symptoms to motor symptoms；

| **Supplement Table 6 Mediation model of RBD(RBDQ-HK) mediating the effects of multiple comorbid negative symptoms on movement disorders (MDS-UDPRS Part III) after controlling for age, disease duration, and education** | | | | | |
| --- | --- | --- | --- | --- | --- |
|  | **Path Coefficients*** | | | | |
|  | **Path a** | **Path b** | **Path ab** | **Path c’** | **Path c** |
| **number of negative symptoms** | β=3.340;  95%(0.847to5.805);  SE=9.308;  P=0.008; | β=0.005  95%(-0.115to0.1424); SE=0.060;  P=0.941; | β=0.015;  95%(-0.369to0.468);  Boot SE=0.204; | β=3.317;  95%(1.415to5.218);  SE=0.962;  p<0.001; | β=3.332; 95%(1.479to5.184);  SE=0.938;  p<0.001; |

Number of negative symptoms (0–3; demoralization,apathy and depression);

Path a ：Association between the number of negative symptoms and RBD；

Path b ：Association between the number of RBD and motor symptoms；

Path ab ：The indirect effect of the number of negative symptoms to motor symptoms；

Path c’：The direct effect of the number of negative symptoms to motor symptoms；

Path c ：The total effect of the number of negative symptoms to motor symptoms；

| **Supplement Table 7Mediation model of EDS(ESS) mediating the effects of multiple comorbid negative symptoms on movement disorders (MDS-UDPRS Part III) after controlling for age, disease duration, and education** | | | | | |
| --- | --- | --- | --- | --- | --- |
|  | **Path Coefficients*** | | | | |
|  | **Path a** | **Path b** | **Path ab** | **Path c’** | **Path c** |
| **number of negative symptoms** | β=1.156;  95%(0.562to1.750);  SE=0.301;  p<0.001; | β=0.196;  95%(-0.299to0.690); SE=0.250;  P=0.436; | β=0.226;  95%(-0.348to0.870);  Boot SE=0.305; | β=3.106;  95%(1.164to5.047);  SE=0.983;  P=0.002; | β=3.332; 95%(1.479to5.184);  SE=0.938;  p<0.001; |

Number of negative symptoms (0–3; demoralization,apathy and depression);

Path a ：Association between the number of negative symptoms and EDS；

Path b ：Association between the number of EDS and motor symptoms；

Path ab ：The indirect effect of the number of negative symptoms to motor symptoms；

Path c’：The direct effect of the number of negative symptoms to motor symptoms；

Path c ：The total effect of the number of negative symptoms to motor symptoms；
